# Sex Hormone Therapies and Alzheimer's Disease: Prevention vs Treatment

**DOI:** 10.1002/alz70855_096003

**Published:** 2025-12-23

**Authors:** Roberta Diaz Brinton

**Affiliations:** ^1^ University of Arizona, Tucson, AZ, USA

## Abstract

**Background:**

Two‐thirds of persons living with Alzheimer's are women. Further, the impact of APOE‐e4 on risk of Alzheimer's is greater in women which is particularly evident in heterozygous women carrying one APOE‐e4 allele. Greater risk of Alzheimer's in women is partially explained by ∼4.5 years greater longevity of women relative to men but is also impacted by the midlife menopausal transition which can influence the other greatest risk factor for AD, aging. Evidence indicates that the disease can begin earlier in women, during the midlife transition of the menopause.

**Method:**

Molecular, cellular, systems biology and medical informatic analyses investigating the impact of estrogen, progesterone and menopausal hormone therapy on neural mechanisms relevant to Alzheimer's disease and progression.

**Result:**

Estrogen promotes molecular, cellular and systems of biology required for glucose metabolism, mitochondrial function, ATP generation and neuroimmune function. Loss of estrogen during menopausal transition initiates an adaptive response which can lead to generation of Alzheimer's‐associated pathology. In preclinical studies, estrogen replacement therapy prevents the shift to auxiliary fuels and associated neurodegenerative pathology. Medical informatic analyses of real‐world menopausal hormone therapy (MHT) for menopause‐related symptoms indicates a significant reduction in risk of Alzheimer's disease. In contrast, both observational and interventional clinical studies of MHT in women without menopausal symptoms have yielded variable outcomes ranging from beneficial to neutral to detrimental.

**Conclusion:**

Key factors influencing MHT efficacy to sustain brain health are stage of the menopausal transition when MHT is initiated and its formulation. MHT initiated early in the menopausal transition for menopausal symptoms has the strongest evidence for sustaining cognitive function and reducing Alzheimer's risk. In contrast, administration of MHT after the menopausal transition is associated with modest increased risk of Alzheimer's. MHT formulation, particularly the progestogen component, can significantly impact efficacy on neural outcomes. Overall, the data are consistent with efficacy of MHT to sustain brain health not to reverse disease. Precision Menopausal Hormone Therapy is an unmet need in women's brain health which has the potential to alter the risk profile in those at greatest risk for Alzheimer's. Research supported by National Institute of Aging, Alzheimer's Association and ADDF.

